# Distinguishing Tuberculosis from Nontuberculous Mycobacteria Lung Disease, Oregon, USA

**DOI:** 10.3201/eid1703.101164

**Published:** 2011-03

**Authors:** Brian A. Kendall, Cara D. Varley, Dongseok Choi, P. Maureen Cassidy, Katrina Hedberg, Mary A. Ware, Kevin L. Winthrop

**Affiliations:** Author affiliations: University of Utah, Salt Lake City, Utah, USA (B.A. Kendall);; Oregon Health and Science University, Portland, Oregon, USA (C.D. Varley, D. Choi, K.L. Winthrop);; Oregon Public Health Division, Portland (P.M. Cassidy, K. Hedberg);; Multnomah County Health Department, Portland (M.A. Ware)

**Keywords:** Bacteria, Mycobacterium tuberculosis, Mycobacterium avium, tuberculosis and other mycobatcteria, pulmonary, respiratory infections, nontuberculous mycobacteria, infection control, diagnosis, dispatch

## Abstract

To determine whether tuberculosis (TB) and nontuberculous mycobacteria (NTM) infection patients could be distinguished from one another with limited information, we compared pulmonary TB and NTM patients during 2005–2006. Our finding that age, birthplace, and presence of chronic obstructive pulmonary disease could differentiate TB and NTM disease could assist tuberculosis control efforts.

Patients seeking treatment who have respiratory specimens positive for acid-fast bacilli present a public health dilemma. Although *Mycobacterium tuberculosis* and nontuberculous mycobacteria (NTM) cause chronic lung infections, only tuberculosis (TB) spreads from person to person by inhalation of organisms expectorated into the air. NTM infections are acquired directly from the environment, where they are often present in soil and various water sources. The prevalence of NTM disease is reported to be increasing and is likely greater than that of TB in the United States (*1**–**3*). Because definitive identification of mycobacterial species can take several weeks, the ability to quickly distinguish NTM from TB on clinical grounds could help public health officials make decisions regarding contact investigations and isolation. To date, little population-based data exist that compare characteristics of pulmonary TB and NTM patients because previous studies have been limited to single institutions (*4**–**6*).

## The Study

We identified patients reported to the Oregon Health Division with pulmonary TB during 2005–2006 who lived within the Portland metropolitan region (Clackamas, Multnomah, and Washington Counties). This region had a combined population of ≈1.55 million in 2005–2006 (*7*). In 2000, the predominant ethnicity in this region was white (75.8%), followed by Hispanic or Latino (11.4%), Asian (6.3%), and black (3.6%), and 11.9% of the population had been born outside the United States (*7*). From a statewide surveillance project, we identified all tri-county residents with NTM respiratory isolates obtained during the same period and then used pulmonary NTM disease criteria of the American Thoracic Society/Infectious Diseases Society of America to define cases of pulmonary NTM disease (*3**,**8*). For each pulmonary TB and NTM case-patient within the tri-county region, we collected demographic information. From physician records, we collected clinical data. We conducted this project under the authority of the Oregon Administrative Rules for special studies to control a public health problem.

We used SAS version 9.1 (SAS Institute Inc., Cary, NC, USA) to compare categorical variables in univariate fashion by the χ^2^ or Fisher exact tests. We calculated the relative proportion (RP) of TB patients with each risk factor compared to the proportion of NTM patients with the risk factor. We used the Student *t* test to evaluate continuous variables. We considered factors with a p value <0.2 for multivariate logistic regression and performed stepwise backward elimination of variables not reaching levels of statistical significance (p<0.05). Using significant variables from our multivariate model, we calculated the positive predictive value (PPV) and 95% exact binomial confidence intervals (CIs) of variables, alone and in combination, for distinguishing TB from NTM disease. Age was dichotomized (<50 and >50 years) based on the age of NTM case-patients to simplify calculation of PPV (*9*).

Eighty-two pulmonary TB patients were reported; all but 2 had complete clinical records for review. We identified 407 patients with respiratory NTM isolates. Clinical records were present for 283 (69.5%) of these patients, of whom 127 (44.9%) met clinical criteria of the American Thoracic Society/Infectious Diseases Society of America for pulmonary NTM disease (*8*). Fifty-four patients lacked information on country of birth. In patients for whom smear data was available, no important difference was found in proportion of case-patients with smear-positive results (38/79 [46%] of TB case-patients vs. 28/47 [60%] of NTM case-patients). In comparison to NTM case-patients, TB patients were younger (median age 44 years, range 5–86 years vs. 67 years, range 12–92 years; p<0.01), more likely to be male (RP 1.6 , 95% CI 1.2–2.2, p<0.01), and more likely to have been born outside the United States (RP 4.0, 95% CI 2.5–6.3, p<0.01) (Table 1). *Mycobacterium avium-intracellulare* complex was the most common etiologic agent of NTM disease in our cohort (114 [90%]).

**Table 1:** Demographic, clinical, and radiographic features of TB patients compared with NTM patients, Oregon, USA, 2005–2006*

Characteristic	No. (%) TB patients, n = 80†	No. (%) NTM patients, n = 127	Relative proportion (95% CI)	p value
Demographics				
Median age, y (range)	44 (5–86)	67 (12–92)	0.95 (0.93–0.96)	<0.01‡
Male	49 (61)	48 (38)	1.6 (1.2–2.2)	<0.01
Not US born§	65 (81)	15 (19)	4.0 (2.5–6.3)	<0.01‡
Clinical signs and symptoms				
Cough	58 (73)	98 (77)	0.9 (0.8–1.1)	0.45
Hemoptysis	12 (15)	28 (22)	0.7 (0.4–1.3)	0.21
Constitutional symptoms¶	56 (70)	61 (48)	1.5 (1.2–1.8)	0.03‡
Chest radiograph				
Bronchiectasis	2 (3)	6 (5)	0.5 (0.1–2.6)	0.71
Cavity	18 (23)	11 (9)	2.7 (1.3–5.3)	<0.01
Effusion	10 (13)	8 (6)	2.1 (0.9–5.0)	0.10
Infiltrate	68 (87)	69 (54)	1.6 (1.3–1.9)	<0.01‡
Lymphadenopathy	4 (5)	3 (2)	2.2 (0.5–9.4)	0.43
Concurrent conditions				
Immunosuppressive medications#	8 (10)	34 (27)	0.4 (0.2–0.8)	<0.01
COPD	2 (3)	29 (23)	0.1 (0.0–0.4)	0.19‡
Previous TB	3 (4)	13 (10)	0.4 (0.1–1.2)	0.11
Diabetes	10 (13)	8 (6)	2.0 (0.8–4.8)	0.12
Tobacco smoking (previous or current)	26 (33)	53 (42)	0.8 (0.5–1.1)	0.19
Lung cancer	4 (5)	8 (6)	0.8 (0.2–2.6)	0.77
HIV/AIDS	0	4 (3)		0.30

*TB, tuberculosis; NTM, nontuberculous mycobacteria; CI, confidence interval; COPD, chronic obstructive pulmonary disease. †Two TB patients excluded because of missing clinical data, 4 from multivariate analysis (n = 78). ‡p value from multivariate analysis including COPD, age, not US born, constitutional symptoms, and infiltrate on radiograph. §54 (26%) patients excluded because of missing country of origin. ¶Fever, night sweats, weight loss, or appetite loss. #Systemic corticosteroids, inhaled corticosteroids, disease-modifying anti-rheumatic drugs, tissue necrosis factor-α inhibitors, cancer chemotherapy, and calcineurin inhibitors.

Clinically, TB patients were more likely to report constitutional symptoms (56 [70%] vs. 61 [48%], RP 1.5, 95% CI 1.2–1.8, p<0.01), less likely to have chronic obstructive pulmonary disease (COPD) (2 [3%] vs. 29 [23%], RP 0.1, 95% CI 0.0–0.4, p<0.01]), and less likely to be using immunosuppressive medications than NTM patients (8 [10%] vs. 34 [27%], RP 0.4, 95% CI 0.2–0.8, p<0.01) (Table 1). The most common immunosuppressive medications were systemic corticosteroids (30 patients [14%]). Patients with TB were more likely to have cavitation (18 [23%] vs. 11 [9%], RP 2.7, 95% CI 1.3–5.3, p<0.01) and infiltrate reported (68 [87%] vs. 69 [54%], RP 1.6, 95% CI 1.3–1.9, p<0.01) on chest radiograph (Table 1).

Birth outside the United States (odds ratio [OR] 26.3, 95% CI 9.9–69.6, p<0.01), constitutional symptoms (OR 3.0, 95% CI 1.1–8.0, p = 0.03), and infiltrate on chest radiograph (OR 7.8, 95% CI 2.6–23.9, p<0.01) were significantly associated with TB in multivariate analysis. Age was inversely related to the likelihood of having TB with an OR of 0.95 (95% CI 0.93–0.98, p<0.01) for each year increase in age. Because of its clinical significance, COPD (OR 0.3, 95% CI 0.1–1.7, p = 0.19) was maintained in the multivariate model. Four patients with missing covariate data were excluded (Table 1).

In our predictive model, age <50 years and birth outside the United States together were highly predictive for TB (PPV 0.98, 95% CI 0.88–1.0). COPD was poorly predictive of TB (PPV 0.06, 95% CI 0.01–0.21). Age >50, US-born status, and COPD together had a PPV for TB of 0.08, 95% CI 0.00–0.38 (Table 2; Figure).

**Table 2:** PPVs of patient characteristics for tuberculosis in Oregon, USA, an area of low tuberculosis incidence, 2005–2006*

Variable	No. patients	No. TB cases	PPV for TB (95% CI)
Age <50 y, not US born	44	43	0.98 (0.88–1.00)
Age >50 y, US born, COPD	12	1	0.08 (0.00–0.38)
COPD	31	2	0.06 (0.01–0.21)
Age <50 y	70	49	0.70 (0.58–0.80)
Not US born	80	65	0.81 (0.71–0.89)
Infiltrate	137	68	0.50 (0.41–0.58)
Constitutional symptoms	117	56	0.48 (0.39–0.57)

*PPV using all patients (n = 207); tuberculosis cases = 80. PPV, positive predictive value; TB, tuberculosis; CI, confidence interval; COPD, chronic obstructive pulmonary disease.

**Figure:**
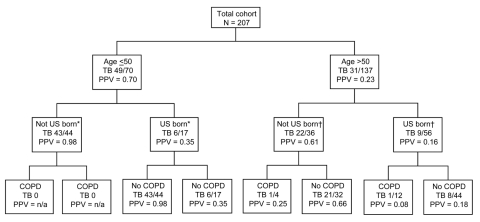
Positive predictive values (PPV) for tuberculosis of demographic and clinical factors in combination. TB, tuberculosis; COPD, chronic obstructive pulmonary disease; *9 patients missing birthplace; †45 patients missing birthplace.

## Conclusions

In this population-based study comparing the demographic and clinical features of TB and NTM patients in a region of low TB incidence, we found that birthplace outside the United States, age, and the presence of COPD can accurately categorize 98% of patients in whom NTM disease is suspected. This information could be useful in making early isolation and treatment decisions in regions of low TB incidence.

According to recent surveillance data from the Centers for Disease Control and Prevention, 26 states had TB incidence similar to Oregon at <3 patients per 100,000 population; nationwide, 59% of patients were born outside the United States (*10*). With regard to the proportion of patients who were not born in the United States, and the proportion of the general population who were not born in the United States, Oregon is similar to many other states with a low-incidence of TB. Fourteen states with low TB incidence have >50% of TB cases occurring in non–US-born patients in a setting in which <12.3% of the total population is not born in the United States. Furthermore, 35 states had a similar racial composition to Oregon with a white, non-Hispanic population >72.6% (*7*). Oregon is therefore representative of many low-incidence TB areas within the United States.

Although the strength of this study is the population-based data, this circumstance also leads to limitations. The ratio of TB to NTM prevalence in a given geographic area likely varies, which affects the degree to which our results can be generalized. Unfortunately, NTM disease prevalence rates are largely unknown. Marras et al. reported a similar prevalence of NTM isolation in Ontario, Canada, but a higher incidence of TB (*11*). They also reported finding fewer *M. avium* complex and more *M. xenopi* and rapidly-growing mycobacteria (*11*). Regions less dominated by *M. avium* complex or with differing TB/NTM prevalence ratios might find different associations. Additionally, further analysis of patients with smear-positive results was precluded by inadequate sample size. A subgroup analysis of smear positive patients in a larger cohort would be useful.

In summary, we found that TB and NTM could be reliably differentiated by determining patient’s birthplace, age, and presence of COPD. Until improved tools are developed for rapid mycobacterial diagnosis, these data might enable public health practitioners and clinicians in other regions with low TB incidence to plan more effective TB control efforts.
